# A randomised trial and economic evaluation of the effect of response mode on response rate, response bias, and item non-response in a survey of doctors

**DOI:** 10.1186/1471-2288-11-126

**Published:** 2011-09-05

**Authors:** Anthony Scott, Sung-Hee Jeon, Catherine M Joyce, John S Humphreys, Guyonne Kalb, Julia Witt, Anne Leahy

**Affiliations:** 1 Melbourne Institute of Applied Economic and Social Research, Faculty of Business and Economics The University of Melbourne Level 7, Alan Gilbert Building, 161 Barry Street, Carlton, VIC 3053, Australia; 2 Department of Epidemiology and Preventive Medicine School of Public Health and Preventive Medicine Monash University, Alfred Hospital Melbourne 3004, Australia; 3 Monash University School of Rural Health PO Box 666 Bendigo Central VIC 3552, Australia; 4 Department of Economics 501 Fletcher Argue Building University of Manitoba, Winnipeg, MB R3T 5V5 Canada

## Abstract

**Background:**

Surveys of doctors are an important data collection method in health services research. Ways to improve response rates, minimise survey response bias and item non-response, within a given budget, have not previously been addressed in the same study. The aim of this paper is to compare the effects and costs of three different modes of survey administration in a national survey of doctors.

**Methods:**

A stratified random sample of 4.9% (2,702/54,160) of doctors undertaking clinical practice was drawn from a national directory of all doctors in Australia. Stratification was by four doctor types: general practitioners, specialists, specialists-in-training, and hospital non-specialists, and by six rural/remote categories. A three-arm parallel trial design with equal randomisation across arms was used. Doctors were randomly allocated to: online questionnaire (902); simultaneous mixed mode (a paper questionnaire and login details sent together) (900); or, sequential mixed mode (online followed by a paper questionnaire with the reminder) (900). Analysis was by intention to treat, as within each primary mode, doctors could choose either paper or online. Primary outcome measures were response rate, survey response bias, item non-response, and cost.

**Results:**

The online mode had a response rate 12.95%, followed by the simultaneous mixed mode with 19.7%, and the sequential mixed mode with 20.7%. After adjusting for observed differences between the groups, the online mode had a 7 percentage point lower response rate compared to the simultaneous mixed mode, and a 7.7 percentage point lower response rate compared to sequential mixed mode. The difference in response rate between the sequential and simultaneous modes was not statistically significant. Both mixed modes showed evidence of response bias, whilst the characteristics of online respondents were similar to the population. However, the online mode had a higher rate of item non-response compared to both mixed modes. The total cost of the online survey was 38% lower than simultaneous mixed mode and 22% lower than sequential mixed mode. The cost of the sequential mixed mode was 14% lower than simultaneous mixed mode. Compared to the online mode, the sequential mixed mode was the most cost-effective, although exhibiting some evidence of response bias.

**Conclusions:**

Decisions on which survey mode to use depend on response rates, response bias, item non-response and costs. The sequential mixed mode appears to be the most cost-effective mode of survey administration for surveys of the population of doctors, if one is prepared to accept a degree of response bias. Online surveys are not yet suitable to be used exclusively for surveys of the doctor population.

## Background

Surveys of medical practitioners can provide important policy-relevant data and information that is often not captured by administrative data or registration databases. There is some suggestion that response rates for surveys of medical practitioners may be falling, with important implications for statistical inference, and for the extent to which results can be generalised and used to inform policy [1-3].

There is growing evidence in the literature about the most effective interventions to increase response rates in general population and doctor surveys. Interventions to improve response rates include incentive-based approaches (e.g. money, gifts, lottery and prize draws), design-based approaches (e.g. survey length, follow-up, content) and mode of administration (e.g. paper, internet, interview). Three key factors that influence doctors' decisions to complete a survey are the opportunity cost of their time; their trust that the results will be used appropriately; and the perceived relevance of the survey [4].

Although the literature about factors influencing response rates is growing, there are three important gaps that this paper aims to address: i) a lack of evidence on the use of mixed mode survey designs; ii) a lack of evidence examining response bias and item non-response, in addition to response rate, and iii) a lack of evidence on the cost-effectiveness of different strategies.

The use of online and web-based surveys is growing, including those where email is the method of contact. Web-based surveys may seem attractive as there are no printing or data-entry costs, but response bias may be an issue, particularly if older respondents are less likely to respond, and a lack of trust in the security of transmitting information over the internet may reduce response rates and increase item non-response [2]. For doctors, emailed surveys have resulted in lower response rates than mailed surveys [4]. This is also reflected in the use of email in non-doctor populations, where meta analyses have found that web-based surveys, mostly using email contact, have a 10-11% lower response rate compared to other modes [5,6]. This is despite the fact that email surveys that include a weblink reduce the number of steps (and time) to complete a survey. However, evidence also shows that email contact can be impersonal and reduce response rates [7]. For doctors, there is an issue of whether the email will reach the respondent or be initially read by administrative staff who may not forward such emails to respondents, though this may also be an issue if mailed surveys are posted to their work address.

Furthermore, the use of different types of mixed mode surveys for doctors has not yet been investigated thoroughly [4,8]. This is important if, for example, younger respondents are more likely to respond to an online survey, whilst older respondents are more likely to respond to a mailed survey. Accounting for doctors' preferences about which survey mode to complete may be important. For example, in a survey of doctors in the United States, paper surveys were preferred to email surveys when they were given the choice [9], and family physicians preferred mail surveys compared to surgeons [10]. The ability of doctors to choose their preferred mode of response to fit with their busy schedules is likely to be important [4,11]. Evidence from non-doctor populations suggests that offering a choice of mode does not increase response rates, but that the sequencing or switching of modes (e.g. paper followed by online) may matter [12-14]. A paper examining this for US physicians showed that mail first, followed by a web survey, had a higher response rate than web followed by mail [8].

Different modes of administration may also influence survey response bias (whether those responding are representative of the population) and item non-response (the extent to which all questions have been completed) as well as overall survey response rates. Response rates are frequently regarded as sentinel indicators of methodological quality in general, and representativeness in particular [15]. Although response rates are often used as a 'conventional proxy' for response bias, there is in fact no necessary relationship between response rate and response bias [16-19]. Despite this, less than half (44%) of published surveys of doctors discuss response bias, and only 18% provided some analysis of it [20]. Item non-response is also an issue, with respondents less likely to answer sensitive questions and some skipping whole sections, depending on how the survey has been designed and administered [21]. High item non-response was found in a web survey, compared to a face-to-face survey, of university students [22], whilst health professionals who were younger, male, and worked in hospitals were more likely to complete a web survey than a mailed survey [23].

There is also a lack of rigorous evidence on the cost-effectiveness of the many different approaches to improve response rates and reduce bias [24]. Email and web surveys may seem cheaper than mailed surveys, and the effects on costs for mixed mode surveys are less clear. Researchers often have limited resources and adoption of all possible measures to increase response rates is usually not possible due to cost constraints and ethical considerations, especially when the study population or sample is widely dispersed. For these reasons, researchers must make choices as to which method leads to the largest increase in response rate (or other outcome) for each dollar spent. For example, up-front financial incentives may be the most effective, but are also costly compared with other approaches [7,25-27]. Baron et al examined the effect of a lottery for GPs in Canada and found a 6.4% increase in the response rate at a cost of $CAD16 per additional returned survey [28]. Bjertnaes et al examined the effects and costs of the number of reminders in a survey of Norwegian physicians, and found that costs per response increased dramatically with telephone follow up [29]. Erdogan and Baker (2002) examined costs and effects of different methods of follow-up in a sample of advertising agency executives, but compared average costs and effect rather than incremental costs and effect [30]. A study that compared the costs of a mail and email survey in a group of academics found the email survey's costs were lower but that mail had a 12% higher response rate [31].

**Figure 1:**
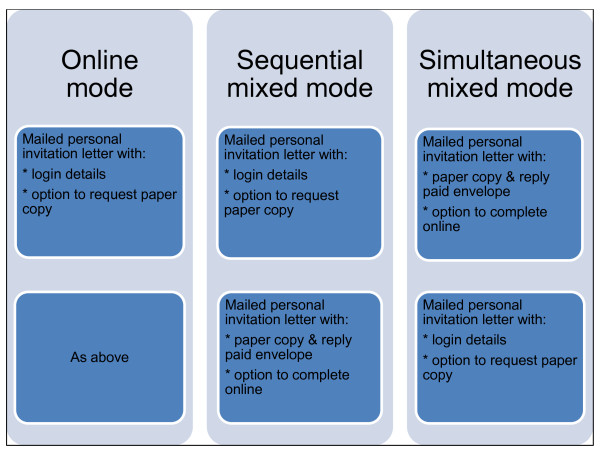
**Description of mode of initial contact and follow up contact for the three arms of the trial**.

The aim of this study is to conduct a randomised trial and economic evaluation of an online survey compared to two types of mixed mode. Our choice of modes reflects the importance to doctors of being able to choose which mode to fill out, and the importance of a personalised mailed letter sent to their preferred mailing address (rather than their work address) as the main mode of contact rather than email. In all three modes, our method of contact was by mailed personalised letter. Three response modes were compared (Figure 1): (i) *Online mode*: a mailed personal invitation letter asked doctors to logon to a secure website to fill out an online version of the questionnaire. Respondents could request a paper copy by phone/fax/email or they could print out a paper questionnaire after they logged on to the website. They were sent a reminder letter around three weeks later that again included login details. (ii) *Sequential mixed mode: *as above, but a paper questionnaire and reply-paid envelope was included with the reminder letter three weeks later; and (iii) *Simultaneous mixed mode: *a paper questionnaire and reply-paid envelope was sent out with the invitation letter, which also contained login details and so respondents could alternatively choose to fill out the survey online if they wished. A reminder letter was sent three weeks later with login details only and no paper survey. Primary outcome measures were response rate, survey response bias and item response. An economic evaluation comparing the costs of each mode of administration was also conducted by applying the results from the trial to the expected costs of the full main wave survey.

Our hypotheses are that:

1). online mode will result in a lower response rate and higher item non-response, compared with the two mixed modes;

2). sequential mixed mode will have a higher response rate than simultaneous mixed mode;

3). the costs of the online mode will be lower than the two mixed modes; and

4). the costs of the sequential mixed mode will be lower than the simultaneous mixed mode.

## Methods

A randomised trial was conducted as part of the third and final pilot survey for Wave 1 of the Medicine in Australia: Balancing Employment and Life (MABEL) longitudinal cohort/panel study of the dynamics of the medical labour market in Australia, focusing on workforce participation and its determinants among Australian doctors [32]. The first wave of data collection, establishing the baseline cohort for the study, was undertaken in 2008.

The questionnaire included eight sections: job satisfaction, attitudes to work and intentions to quit or change hours worked; a discrete choice experiment (DCE) examining preferences and trade-offs for different types of jobs; characteristics of work setting (public/private, hospital, private practice); workload (hours worked, on-call arrangements, number of patients seen, fees charged); finances (income, income sources, superannuation); geographic location; demographics (including specialty, qualifications, residency); and family circumstances (partner and children). There were four versions of the survey, each differing slightly in order to tailor them to the type of doctor: GPs, specialists, specialists-in-training, and hospital non-specialists. Although survey length also matters for response rates, the context of the survey and research questions being tested required a long questionnaire, to ensure that sufficient data were collected to adequately test study hypotheses [32]. The length ranged from 58 questions in an eight-page booklet (for specialists-in-training), to 87 questions in a 13-page booklet (for specialists). In all modes, doctors in remote and rural areas (defined using the Rural, Remote and Metropolitan Area (RRMA) classification to doctors in RRMA 6 (Remote centre with population > 5,000) and RRMA7 (Other remote centre with population < 5,000)), mainly GPs, were given a cheque for $100 that was enclosed with the invite letter to recognise both their importance from a policy perspective, and the significant time pressures on these doctors. The purpose was to draw meaningful inferences about recruitment and retention in rural and remote areas. Pre-paid monetary incentives, not conditional on response, have been shown to double response rates [25]. The survey described in this paper was also the main pilot survey for the main wave of MABEL, so it was important to pilot the administration of these incentives. However, they did not influence the outcome of this trial as randomisation ensured approximately equal numbers of cheques going out in each arm of the trial.

The process of logging in and completing the survey online was kept as simple as possible. Users were directed to the main web page (http://www.mabel.org.au), where they clicked on the 'Login' link, which directed them to a login page where they entered their username and password. They were then directed to the first page of the survey. Respondents could save their responses and logout, and then login again to complete the survey, and they could skip questions. Once logged in, the padlock icon was visible, indicating that the website was secure.

The primary outcomes of interest in the trial were response rates, survey response bias with respect to age, gender, doctor type and geographic location, and item-response (the percentage of completed items). A three-arm parallel trial design was used with equal randomisation across arms. The sample size for the trial was calculated to detect a difference of 5% in the response rate at the 95% level of statistical significance, and with a power of 80%. This indicated that a sample of 900 doctors in each arm of the trial would be required, 2,700 doctors in total. This represented just under 5% (2700/54,160 = 0.04985) of all doctors undertaking clinical practice on the Australian Medical Publishing Company's (AMPCo) Medical Directory, which includes all doctors in all States and Territories of Australia and formed our sampling frame. This national database is used extensively for mailing purposes (e.g. the Medical Journal of Australia). The Directory is updated regularly using a number of sources. AMPCo receives 58,000 updates to doctors' details per year, through biannual telephone surveys, and checks medical registration board lists, Australian Medical Association membership lists and Medical Journal of Australia subscription lists to maintain accuracy. The directory contains a number of key characteristics that can be used for checking the representativeness of the sample and to adjust for any response bias in sample weighting. These characteristics include age, gender, location, and job description (used to group doctors into the four types).

A 4.9% stratified random sample of doctors was therefore taken, with stratification by four doctor types (general practitioners (GPs), specialists, doctors enrolled in a specialist training program, and non-specialist hospital doctors (including interns and salaried medical officers)), and six rural/remoteness categories (Rural, Remote and Metropolitan Area (RRMA) classification). This produced a list of 2,702 doctors. Doctors in this sample were then randomly allocated to a response mode by AS using random numbers generated in STATA. The AMPCo unique identifiers for each of the three groups were sent to AMPCo who conducted the mailing of invitation letters, and survey materials were mailed in late February 2008. Survey invitation letters indicated the University of Melbourne and Monash University as responsible for the survey. AMPCo also provided individual-level data on the population of doctors so we could examine response bias. Doctors were aware of which mode they had been allocated to on receipt of the invitation letter. The survey manager (AL) recorded responses and organised data entry and was blinded to group allocation. SH analysed the data and was not blinded to group allocation. Analysis was by 'intention to treat'.

Analysis included comparisons of response rates; estimation of means and proportions of respondents by age, gender, doctor type, and geographic location compared to the doctor population; logistic regression of response bias; and comparisons of the proportion of missing values (item non-response). The statistical significance of the differences between the response rates of the three response modes was analysed using a probit model with response (0/1) as the dependent variable and two dummy variables for response mode, with online as the reference category. The difference between the sequential and simultaneous modes was tested using the restriction that their coefficients be equal. Although respondents were randomly allocated across modes, it is still important to test whether any specific/particular respondent characteristics influenced the response rate. The probit model therefore included age, gender, doctor type and geographic location. Survey response bias was examined using a multinomial logit model of respondents (= 1) and the total population of doctors (= 0), with age, gender, geographic location and doctor type as independent variables. For item non-response, a comparison of the proportion of completed items was supplemented using generalised linear models that controlled for differences due to age, gender, geographic location and doctor type [33]. Analysis of geographic location was based on the Australian Standard Geographic Classification (AGSC) Accessibility and Remoteness Index for Australia (ARIA)[34].

The economic evaluation compared the costs of consumables (rental of AMPCo list, printing of surveys, letters, fax forms, further information fliers and reply-paid envelopes, mail-house processing costs, postage and data entry) across each mode of survey administration. The costs of researcher and staff time were the same for each mode, as each mode required the development of both the paper and web survey, and time liaising with AMPCo and the printers. These costs were therefore not included in the comparison of costs between modes. The expected costs for the main wave 1 survey were estimated based on sending out a survey to all doctors on the AMPCo database (n = 54,168) and using the response rates from the randomised trial to estimate the number of respondents. Data on the three primary outcome measures are presented alongside data on costs.

## Results

Responses were received between March and October 2008. The characteristics of doctors in the three groups for the study sample are shown in Table 1. Although there are some small differences of up to three percentage points, the three groups are broadly similar in terms of key characteristics. The comparison of response rates across modes is shown in Table 2. Response rates were between 6 and 7 percentage points higher for the two mixed modes, compared to online (Table 2). Table 3 shows response rates by mode and doctor type. Specialists had the highest overall response rates and GPs the lowest. Response rates for simultaneous and sequential mixed modes were between 2 and 6 percentage points higher for GPs, and between 10 and 15 percentage points higher for specialists. For hospital non-specialists, the response rate for simultaneous mixed mode was four percentage points higher than online, but four percentage points lower than sequential mixed mode. For specialists in training, the simultaneous mixed mode had the lowest response rate, with the sequential and online modes producing similar results.

**Table 1:** Group characteristics of full sample

	Simultaneous mixed	Sequential mixed	Online	Total
	N (%) 900	N (%) 900	N (%) 902	N (%) 2,702
**Doctor type**				
GPs	334 (37.1)	388 (43.1)	369 (40.9)	1,091 (40.3)
Specialists	333 (37.0)	318 (35.3)	317 (35.1)	968 (35.8)
Hospital non-specialists	161 (17.9)	140 (15.6)	142 (15.7)	443 (16.4)
Specialist in training	72 (8.0)	54 (6.0)	74 (8.2)	200 (7.4)
				
**Geographic location^1^**				
Major city	739 (82.1)	711 (79)	745 (82.6)	2,195 (81.2)
Inner regional	116 (12.9)	140 (15.6)	110 (12.2)	366 (13.6)
Outer regional	42 (4.7)	40 (4.4)	43 (4.8)	125 (4.6)
Remote	1 (0.1)	5 (0.6)	2 (0.2)	8 (0.3)
Very remote	2 (0.2)	4 (0.4)	2 (0.2)	8 (0.3)
				
**Age Group**				
< = 30	65 (7.2)	64 (7.1)	58 (6.4)	187 (6.9)
30-39	164 (18.2)	150 (16.7)	157 (17.4)	471 (17.4)
40-49	191 (21.2)	200 (22.2)	225 (24.9)	616 (22.8)
50-59	210 (23.3)	212 (23.6)	205 (22.7)	627 (23.2)
60-69	103 (11.4)	123 (13.7)	100 (11.1)	326 (12.1)
70 < =	41 (4.6)	47 (5.2)	44 (4.9)	132 (4.9)
missing	126 (14.0)	104 (11.6)	113 (12.5)	343 (12.7)
				
**Gender**				
Female	280 (31.1)	308 (34.2)	289 (32.0)	877 (32.4)
Male	620 (68.9)	590 (65.6)	613 (68.0)	1,823 (67.5)

1. Australian Standard Geographical Classification (ASGC) classification Remoteness Areas [34].

**Table 2:** Response rates by mode of administration

	All doctors	Simultaneous mixed	Sequential mixed	Online
**a) Total**	2,702	900	900	902
b) Useable responses (with at least one question answered)	476	175	185	116
c) Refusal (i.e. paper copy returned blank, declined)	11	5	4	2
d) No contact (return to sender)	58	18	15	25
e) No responses	2,133	690	690	753
f) Not eligible (i.e. retired, no longer in clinical practice)	24	12	6	6

Response rate (b/(a-f))	17.77%	19.71%	20.69%	12.95%

Contact rate ((b+c+e))/(a-f))	97.83%	97.97%	98.32%	97.21%

**Table 3:** Response rates by mode of administration and doctor type^1 ^(in %)

	Simultaneous mixed	Sequential mixed	Online	Total
GPs	16.01	19.22	13.62	16.34
Specialist	26.23	22.86	11.18	20.17
Hospital non-specialists	16.77	20.71	12.68	16.70
Specialist in training	13.89	18.52	17.57	16.50

1. Response rate for those eligible to respond.

The difference in response rates across modes was statistically significant (Table 4). The table reports the marginal effects of each response mode compared with the online response, and can be interpreted as percentages. Controlling for other factors, the simultaneous mixed mode had a response rate 7 percentage points higher than online, and the sequential mixed mode was 7.7 percentage points higher than online. The effect of sequential mixed mode was not significantly different from simultaneous mixed mode (χ^2 ^= 0.16, p = 0.69). Specialists were 16 and 13 percentage points more likely to respond to the simultaneous and sequential mixed modes respectively, than to the online mode. Differences for other types of doctor were not statistically significant. The probit model also controls for differences in age, gender, doctor type and geographic area on response rate. Overall, females were less likely to respond, and the specialists' response rate was 5.3 percentage points higher than GPs. GPs in outer regional and very remote areas were more likely to respond than those in major cities; this was partly due to a $100 financial incentive provided to doctors in outer regional and very remote areas.

**Table 4:** The effect of mode on response rates (probit regression model)

	All doctors	GPs	Specialists	Hospital non-specialists	Specialists in training
	**Marginal effects (standard error)**				

Simultaneous mixed	0.070***	0.028	0.160***	0.034	-0.030
	(0.020)	(0.030)	(0.036)	(0.046)	(0.060)
Sequential mixed	0.077***	0.053	0.133***	0.072	0.013
	(0.020)	(0.028)	(0.037)	(0.049)	(0.065)
< age30	0.074	0.086	-	0.276	0.108
	(0.046)	(0.156)	-	(0.160)	(0.166)
age30-39	0.037	-0.008	0.029	0.229	0.198*
	(0.027)	(0.040)	(0.048)	(0.163)	(0.085)
age50-59	0.054*	0.039	0.058	0.418	0.009
	(0.023)	(0.031)	(0.036)	(0.245)	(0.144)
age60-69	0.002	0.020	-0.018	-	-
	(0.026)	(0.038)	(0.038)		
> = age70	-0.028	-0.063	0.005	-	-
	(0.035)	(0.042)	(0.058)		
Age missing	-0.037	-0.034	-0.080*	0.208	0.127
	(0.025)	(0.036)	(0.039)	(0.190)	(0.125)
Female	-0.043**	-0.029	-0.066	-0.048	-0.080
	(0.017)	(0.024)	(0.036)	(0.037)	(0.056)
Specialist	0.053**	-	-	-	-
	(0.018)				
Hospital non-specialist	-0.013	-	-	-	-
	(0.027)				
Specialist in training	0.009	-	-	-	-
	(0.032)				
Inner Regional	0.025	0.024	0.053	-0.050	0.140
	(0.023)	(0.030)	(0.045)	(0.054)	(0.105)
Outer Regional	0.051	0.119*	-0.056	-	-
	(0.040)	(0.052)	(0.064)		
Remote	0.231	0.354	-	-	-
	(0.182)	(0.249)			
Very Remote	0.517**	0.542**	-	0.459	-
	(0.163)	(0.185)		(0.315)	

N	2702	1091	966	430	196
Log likelihood	-1255	-469	-461	-188	-84
χ^2 ^(df)	59.7*** (16)	28.3** (13)	37.4*** (10)	12.6 (9)	11.3 (8)
Pseudo R^2^	0.03	0.03	0.04	0.04	0.05

Marginal effects for discrete change of dummy variable from 0 to 1; * P < 0.05, ** P < 0.01, *** P < 0.001; standard errors are in parentheses.

Doctors allocated to each mode were given the opportunity to complete the survey online or on paper. Table 5 shows that of those allocated to the simultaneous mixed mode, 21% chose to complete the survey online, whilst of those in the online group only 3% requested and filled out a paper survey. Doctors allocated to the sequential mixed mode group were more likely to fill out a paper survey (62%) than an online one (38%).

**Table 5:** Actual mode of response by allocated survey mode

	Group allocation			
	Simultaneous mixed	Sequential mixed	Online	Total
**Paper returned (%)**	78.86	61.62	3.45	53.78
**Online completed (%)**	21.14	38.38	96.55	46.22

**Total number of surveys**	175	185	116	476

Response bias was examined for each mode by comparing the characteristics of respondents to each mode with the population of all doctors in Australia. This was undertaken using a multinomial logit model with four outcomes: simultaneous, sequential, online and population. Table 6 shows the odds ratios for those comparisons and factors that were statistically significant at the 95% level. For example, both mixed modes showed evidence of response bias, whilst the characteristics of online respondents were the same as the population. Those who filled out the simultaneous mixed mode were twice as likely to be specialists when compared to the population, whilst those filling out the sequential mixed mode were more likely to be older and more likely to be in a non-metropolitan area when compared to the population. The results also show that specialists were 2.4 times more likely to complete the simultaneous mixed mode than the online, and that those aged over 60 and in inner regional areas were more likely to complete the sequential mixed mode than the online mode.

**Table 6:** Response bias for each mode (Odds ratios and 95% CI)^1^

	age50-59	age60-69	Specialist	Inner Regional	Outer/ remote
Simultaneous mixed compared to Population			2.00 (1.39, 2.87)		

Sequential mixed compared to Population	1.79 (1.16, 2.75)	1.67 (1.01, 2.78)		1.49 (1.00, 2.22)	1.73 (1.01, 2.97)

Simultaneous mixed compared to online			2.42 (1.38, 4.26)		

Sequential mixed compared to online		3.22 (1.26, 8.22)		2.13 (1.02, 4.44)	

1. Results from multinomial logit model. Only statistically significant results (P < 0.05) are reported. Full model results available from authors

Item non-response was examined by calculating the average percentage of items completed, and the percentage of respondents who completed all relevant questions, i.e. whether the percentage of items completed = 100% (Table 7). If a question was 'not applicable', this was counted as a completed question. The order of the sections in the survey is reflected in these tables, with the job satisfaction section coming first. Note that the online survey allowed respondents to skip questions, as would be the case in the paper survey. Overall, the online mode shows the lowest average percentage of items completed, with almost 89% of questions answered compared to around 92% for each of the other modes. This is the case for all sub-sections of the survey, with the section on finances, which includes income questions, having the lowest average percentage of items completed of 80%. This difference is statistically significant, as shown in the first half of Table 8, with odds ratios of 1.48 for paper and 1.53 for mixed mode compared to online. Table 8 also shows statistically significant differences for some sections of the survey. The sequential mixed mode was more likely to have a higher percentage of items completed than the online for the sections on DCE, workload, and location. The simultaneous mixed mode was more likely to have a higher percentage of items completed than the online in the 'About You' and 'Family' sections.

**Table 7:** Item response by mode and questionnaire section (%)

	Simultaneous mixed		Sequential mixed		Online		Total	
	***Average Percentage of items completed***	***Percentage with 100% completed items***	***Average Percentage of items completed***	***Percentage with 100% completed items***	***Average Percentage of items completed***	***Percentage with 100% completed items***	***Average Percentage of items completed***	***Percentage with 100% completed items***

Job Satisfaction	99.5	89.7	99.4	88.6	98.5	90.5	99.2	89.5
DCE	94.9	90.3	98.4	96.2	94.8	91.4	96.2	92.9
Work Places	84.6	21.7	86.8	23.2	83.0	22.4	85.1	22.5
Workload	90.3	35.4	91.5	36.8	85.8	44.0	89.7	38.0
Finances	85.8	44.0	85.7	41.1	80.2	44.8	84.4	43.1
Location	92.2	46.9	93.0	50.8	88.6	51.7	91.7	49.6
About You	95.3	80.0	92.8	71.9	88.1	70.7	92.5	74.6
Family	97.9	94.9	96.6	91.4	93.6	85.3	96.4	91.2

Total	92.0	1.1	92.5	2.2	88.5	6.9	91.4	2.9

1. Percentage of items completed: $\sum_{n}\left(\frac{q_{i}}{t}*100\right)/n$; the mean of the percentage of completed questions per respondent *i*, where *q *is the number of completed questions, *t *is the total number of questions, and *n *is the total number of respondents.

**Table 8:** Item non-response by mode (Odds ratio, 95% CI)

	Job Satisfaction	DCE	Work Places	Workload	Finances	Location	About You	Family	Total
**Average Percentage of items completed^1^**									
Simultaneous mixed (compared to online)	2.85	1.10	1.14	1.52	1.48	1.46	2.67**	3.08**	1.48*
	[0.86,9.42]	[0.42,2.88]	[0.83,1.55]	[0.92,2.53]	[0.94,2.33]	[0.98,2.18]	[1.42,5.00]	[1.32,7.20]	[1.03,2.14]
Sequential mixed (compared to online)	2.53	3.83*	1.31	1.70*	1.41	1.55*	1.68	1.87	1.53*
	[0.81,7.91]	[1.14,12.95]	[0.97,1.76]	[1.05,2.76]	[0.90,2.20]	[1.02,2.37]	[0.96,2.94]	[0.87,4.03]	[1.09,2.17]

N	476	476	476	476	476	476	476	476	476.
Log likelihood	-18.397	-63.804	-149.636	-129.201	-169.354	-106.841	-105.201	-63.197	-107.395

**Whether 100% of items were completed^2^**									
Simultaneous mixed (compared to online)	0.95	0.96	0.95	0.67	0.98	0.77	1.75	3.25**	0.13*
	[0.42,2.11]	[0.42,2.19]	[0.53,1.72]	[0.40,1.11]	[0.60,1.59]	[0.48,1.24]	[0.98,3.12]	[1.38,7.65]	[0.02,0.67]
Sequential mixed (compared to online)	0.82	2.64	0.99	0.76	0.84	0.85	1.10	1.86	0.23*
	[0.37,1.81]	[0.97,7.21]	[0.55,1.78]	[0.46,1.26]	[0.51,1.38]	[0.53,1.36]	[0.64,1.87]	[0.90,3.82]	[0.07,0.78]

N	476	476	459	476	476	476	476	476	459
Log likelihood	-156.70	-112.68	-237.51	0.67	-310.12	-320.57	-253.63	-134.05	-51.28

1. Mean proportion of questions completed. A generalised linear model controlling for age, gender, doctor type and rurality.

2. Whether a respondent completed all questions (= 1). Logistic regression model controlling for age, gender, doctor type and rurality * P < 0.05, ** P < 0.01, *** P < 0.001

Although the percentage of questions completed overall was 91.4%, only 2.9% of respondents completed every question, and this was lowest for simultaneous mixed mode (1.1%), followed by sequential mixed mode (2.2%), and was highest for online mode (6.9%) (Table 7). These differences were statistically significant (second half of Table 8), with odds ratios of 0.13 for paper mode and 0.23 for mixed mode compared to online. The proportion of respondents completing all questions was similar across the modes for each section. Those using simultaneous mode were significantly more likely to complete all questions in the 'Family' section compared to those in the online mode (Table 8).

The costs of each mode were estimated for the first wave of the survey, which was to be sent out to the population of doctors in Australia (Table 9). The online mode has the lowest total cost, followed by the sequential mixed mode, with simultaneous mixed mode having the highest cost. The total cost of the simultaneous mixed mode is 38% higher than online, and 21% higher than sequential mixed mode. The sequential mixed mode total costs are 14% higher than the online mode. The main sources of cost differences between modes are related to handling and postage of the mail-out, printing of surveys, and data entry for paper copies.

**Table 9:** Effect of mode on survey costs (2008 prices, in $AU)

	Simultaneous mixed	Sequential mixed	Online
***Total costs***			
AMPCo rental of list	$15,830	$15,830	$15,830
Monetary incentives	$60,000	$60,000	$60,000
Web survey costs	$4,000	$4,000	$4,000
AMPCO handling and postage	$97,157	$72,024	$65,361
Printing of letters, further info, fax sheet, envelopes	$17,981	$18,470	$18,458
Printing of surveys	$21,284	$19,794	$10,995
Data entry	$33,684	$16,630	$6,636
**Total**	**$249,936**	**$206,748**	**$181,280**
			
***Incremental cost effectiveness ratios (compared to online mode)***			
Response rate	19.71%	20.69%	12.95%
Estimated number of responses	10,677	11,208	7,015
Change in response rate compared to online	6.76%	7.74%	-
Change in number of responses compared to online	3,662	4,193	-
Change in total cost compared to online	$68,656	$25,468	-
**Additional cost per 1% increase in response rate^1 ^**	**$10,156**	**$3,290**	-
**Additional cost per additional response^2 ^**	**$18.75**	**$6.07**	-

Notes: 1. Δcost/Δ response rate; 2. Δcost/Δ number of responses

Table 9 shows incremental cost-effectiveness ratios with respect to changes in response rate and number of responses. Comparing sequential and simultaneous mixed modes to online, sequential mixed mode was the most cost-effective relative to online. Costs were $6.07 per additional response, and $AUS3, 290 per 1% increase in the response rate, compared to online. Although the main outcomes were similar for the two mixed modes, the sequential mode was cheaper due to lower printing, mailing and data entry costs. Using the sequential mixed mode resulted in total costs which were 21% lower than for the simultaneous mode, with no detrimental impact on response rate, survey response bias, or item non-response.

## Discussion

This study has compared response rates, survey response bias, item non-response, and costs across three modes of conducting a doctor survey. Mailing a letter inviting respondents to complete the questionnaire online, followed by a mailed reminder letter and paper copy of the survey, was the most cost-effective mode of administration. Although online modes were less costly, due to lower printing and data entry costs, and did not exhibit evidence of increased response bias, the response rates and item completion rates were lower than for the sequential and simultaneous mixed modes. The online mode had lower item completion rates in the sections on the DCE, workload, and personal and family characteristics. Although the sequential mode is the most cost-effective with respect to response rates, whether this is chosen would depend on the weight given to the existence of response bias when compared to the population of doctors.

We find no support for the hypothesis that offering a simultaneous choice of modes results in lower response rates than a sequenced choice of mode. Literature from non-doctor populations suggests that sequencing may be better than simultaneous choice [12,13]. Though there is a small difference in response rates for both mixed modes, this is not statistically significant.

Lower response rates in the online mode arguably reflect the population being surveyed, their familiarity with and trust in the internet, and reliability of access to the internet, especially in remote regions of Australia. Most doctors choose to fill out a paper questionnaire, possibly suggesting that they are less comfortable with doing a survey online or have issues with sending confidential information over the internet. This is reflected in a higher rate of item non-response for most sections of the survey, especially for the more personal questions. This finding occurred despite assurances about confidentiality and the fact that information was being sent over a secure internet connection. Doctors may also prefer the 'portability' of a paper-copy which they can fill out at the office, at home or whilst travelling. Online modes are also becoming more portable (i.e. not confined to the desktop PC) with the use of laptops, touch screen tablets and other mobile devices, so the preference for a paper copy may erode over time. A key issue in relation to survey response is the need to minimise the opportunity cost of survey completion for respondents. The need for internet access and the time it takes to logon needs to be balanced against filling out a paper survey that needs to be posted. A potential reason for the lower online response rate was the need by respondents to find a website and login using their username and password provided in the letter. Once at the website, they had to go to a login page, enter their details, and were then directed to the beginning of the survey. Though this takes time compared to an email survey with an embedded website link, it does provide a more secure process that may have increased the confidence of respondents in the security of the website.

Response rates in all three arms could be regarded as low, an increasing issue for surveys of doctors [1-3]. It is noteworthy that our comparative analysis with the population of Australian doctors showed that the mode with the lowest response rate (online) was the most representative, confirming the point noted in the introduction, that response rate and response bias are separate issues and should both be explicitly analysed to ensure appropriate interpretation.

Our study used a diverse sample of doctors with respect to age, specialty and geographic location, increasing the generalisability of the results. Although the trial was not designed for sub-group analysis, specialists in training allocated to the online mode had a higher response rate (17.5%) than those allocated to the simultaneous mixed mode (13.89%), and a similar response rate to those in the sequential mixed mode (18.52%). For those conducting surveys of younger doctors and doctors in training, who are more likely to be familiar with and trusting of the internet, online surveys may be a more desirable option, though item non-response may be an issue. However, specialists had the highest response rate for the simultaneous version (26.2% compared with 22.9% for mixed) and the lowest response for the online mode (11.2%). The routine use of exclusively online surveys for the population of doctors may therefore be some time off, at least until the current older cohorts have been replaced by younger cohorts.

The unit costs of printing and survey administration are likely to vary across geographic locations and companies, though they are not likely to vary across modes within geographic locations, and so should not influence our findings. Printing costs vary greatly with volume, such that for the pilot online mode the unit cost per printed questionnaire (for those requesting a paper survey) was $AUD5.90. However, the unit cost of printing 54,169 paper questionnaires (for the ensuing main wave survey) was $AUD0.32. The relationship between unit costs and volume printed is not linear. The costs of establishing the online survey will also vary across settings, although there are now many low cost survey packages available that cover most needs, some of which can be re-programmed if necessary.

Our results are in line with other research showing that lower response rates are likely to result from online surveys than mailed surveys [5,6]. Other studies have compared mail and online mixed modes in non-doctor samples [12,13]. However, these studies have not examined costs. There are many different types of response mode, and different combinations of mixed modes, that can potentially be used in surveys of doctors. Further research is required in a number of areas. First, comparisons are needed of modes that offer choice compared to those that do not [14]. Second, all comparisons need to include an examination of the changes in costs. This is mentioned frequently in the literature as a motivation for using online and mixed modes, but there is hardly any evidence of the differences in costs.

## Conclusion

Our study is the first, in the context of a large national survey of doctors, to include an economic evaluation alongside a randomised trial using standardised methods. Of the alternatives compared in our study, the sequential mixed mode had the lowest cost per response compared to online. Decisions on the appropriate response mode will ultimately be a function of the study objectives and context, but for large national surveys of the doctor population that include doctors at different stages of their career, the sequential mixed mode seems to be the preferred option.

## Competing interests

All authors have completed the Unified Competing Interest form at http://www.icmje.org/coi_disclosure.pdf (available on request from the corresponding author) and declare that none of the authors have financial or non-financial interests that may be relevant to the submitted work.

## Authors' contributions

AS, CJ, GK, JH, JW, SJ jointly conceived of this work. All authors participated in designing and developing the study instruments and procedures. AS drafted the paper, and SJ maintained the dataset and conducted the statistical analysis. All authors assisted with re-drafting sections of the paper and interpreting results. AL organised the administration of the survey and data entry. All authors approved the final version of the manuscript. AS is the guarantor.

## Pre-publication history

The pre-publication history for this paper can be accessed here:

http://www.biomedcentral.com/1471-2288/11/126/prepub
